# Special Issue “GPCRs: Ligands and beyond 2022”

**DOI:** 10.3390/ph15060647

**Published:** 2022-05-24

**Authors:** Erika Cione, Maria Cristina Caroleo

**Affiliations:** 1 Department of Pharmacy, Health and Nutritional Sciences, University of Calabria, 87036 Rende, Italy; 2 Department of Health Sciences, University of Magna Graecia-Catanzaro, 88100 Catanzaro, Italy; mariacristina.caroleo@unicz.it

The human genome encodes more than 800 different G protein-coupled receptors (GPCRs), uncovering their importance in human physiology. These integral membrane proteins sense a diverse spectrum of chemical signals in a highly selective way and mediate a wide array of cellular signaling cascades, resulting in a plethora of homeostatic responses. Understanding the ligand recognition, activation, signaling and regulation of these receptors is of primary importance not only to shed light on several aspects of human physiology but also for developing novel therapeutic approaches. Regarding this concern, GPCRs are the target of more than 30% of the drugs on the market. Activated by external stimuli through coupling to different G-proteins or arrestins, GPCRs elicit a cyclic adenosine 3,5-monophosphate response, calcium mobilization or the phosphorylation of extracellular regulated protein kinases 1/2. However, noncanonical interactions between G proteins and GPCRs that do not result in nucleotide exchange and classical G protein signaling are now recognized. These new layers of functional selectivity have greatly expanded the opportunities for therapeutic interventions and the development of new chemical biology tools, such as advanced structural and computational methods, and have further fostered our understanding of GPCRs’ regulatory mechanisms, paving the way for future drugs. In this frame, the recent developments in the field of protein engineering and the crystallography of membrane proteins are considered major advances in GPCR biology. Although the discovery of natural compounds and/or endogenous ligands molecules on GPCRs is still ongoing, the wide availability of ligand-bound GPCR structures has provided crucial insights into the structure, function, and pharmacology of these receptors, empowering the application of structure-based drug design (SBDD) approaches for the discovery of potent candidates with favorable pharmacological profiles. The evidence of new types of ligand–receptor–effector relationships, such as inverse agonism, positive and negative allosterism, multimeric receptor pharmacology, and ligand-biased signaling has further led to novel methods for the therapeutic potential of GPCRs. With this backdrop, the “GPCRs: Ligands and beyond 2022” Special Issue focuses on recent advances in GPCRs’ biology. This thematic issue consists of five original articles and five reviews covering a broad range of topics related to medicinal chemistry and the therapeutic potential of this integral membrane protein family. The first original article from Bassani and coworkers [1] examines the effect of considering the allosteric sodium ion when molecular docking approaches are applied to GPCR antagonists. They demonstrate that a small increment in the docking programs’ performance is observable if the sodium ion is kept during the docking runs just for those crystal structures in which the alkaline ion was resolved, while for the other complexes, the trend is the opposite, favoring the solution of not considering sodium during the docking calculations. With this study, the authors provide new insights into the performance of docking programs when applied to research about GPCR antagonists. A new scientific approach for membrane G-protein analysis is proposed by Wiseman and coworkers [2]. Using databases with available structures of the G protein-coupled receptors (GPCR), the authors demonstrate that geometric morphometrics methodology can be useful to detect, quantify and analyze receptor shape variation in a three-dimensional manner. With this study, the authors introduce a novel system which can also be used as a future tool in sense-checking newly resolved structures and planning experimental design. The work by Mendez-Luna and coworkers [3] is a joint investigation combining computational tools and experimental assays in order to design potential drug candidates inhibiting the G protein-coupled estrogen receptor (GPER). Under this context, the authors design three compounds that showed notable inhibitory activity against GPER when tested in nonconventional cell models. In addition, they identify the key entities (i.e., bromine atom and NH group of the piperidine ring) that could be functionalized to improve the activity of the compounds designed to target this receptor. Ozenil and coworkers [4] focus their studies on the subtype selectivity in order to develop orthosteric mAChR ligands for therapeutic and diagnostic purposes. Within this work, they synthesize chiral hydrobenzoin esters of arecaidine and investigate their properties toward mAChRs through biological and computational approaches. They find that all synthesized stereoisomers behave as antagonists toward mAChR M1. Moreover, they highlight the relevance of chiral ligands in the search for subtype selectivity. The therapeutic potential of bombesin antagonists is instead investigated by Rasaeifar and coworkers [5]. Through a modeling study and in silico screening, using a simple pharmacophore, the authors shed light on the stereochemical requirements for small molecule binders to the BB1 bombesin receptor and identify of a set of small molecules. Overall, the study provides the rationale for designing new experiments and small molecule ligands. The recent evidence on adhesion G protein-coupled receptors (aGPCRs) is instead the focus of the critical review from Hsi-Hsien Lin and colleagues [6]. The authors discuss the possible mechanism of mechanotransduction of aGPCR based on their structural and functional characteristics with a wide and interesting physio-pathological perspective. The next four reviews are thematically connected with a shared focus on GPCRs as therapeutic target. Deepak and colleagues [7] provide an overview of lipid mediators and lipid GPCRs with reference to their role in physiology and diseases and to their value as drug targets. The authors also summarize current advancements in the understanding of structural features of lipid GPCRs and discuss the implications of these findings in the frame of drug discovery and development. Gonçalves-Monteiro and colleagues [8] highlight the emerging biological impact of the nuclear GPCRs. The authors summarize recent evidence on nuclear GPCRs’ expression and function in both physiological and pathological conditions, namely non-communicable disease, thus presenting nuclear GPCRs as novel therapeutic targets. Ayoub and Vijayan [9] provide an overview on pharmacological and functional targeting of G protein-coupled receptors (GPCRs) by hemorphins, short peptides produced by the proteolysis of the beta subunit of hemoglobin and their involvement in physiology and pathophysiology. The authors also discuss the therapeutic value of targeting the hemorphin–GPCR axis in different systems. Finally, Zang and colleagues [10] highlight recent advances in the development of A_2A_ adenosine receptor (A_2A_AR) antagonists for cancer immunotherapy. The authors discuss the therapeutic potential of representative A_2A_AR antagonists in the frame of experimental and clinical data and point out the need for A_2A_AR antagonist drug candidates with high binding affinity and for multiparametric optimization of lead compounds in order to develop clinically useful A_2A_AR antagonists.

We hope the readers enjoy this Special Issue and are inspired to develop new approaches for the rational design of pharmacological GPCR regulators.
